# Diffusion MRI and Novel Texture Analysis in Osteosarcoma Xenotransplants Predicts Response to Anti-Checkpoint Therapy

**DOI:** 10.1371/journal.pone.0082875

**Published:** 2013-12-16

**Authors:** Parastou Foroutan, Jenny M. Kreahling, David L. Morse, Olya Grove, Mark C. Lloyd, Damon Reed, Meera Raghavan, Soner Altiok, Gary V. Martinez, Robert J. Gillies

**Affiliations:** 1 Department of Cancer Imaging and Metabolism, H. Lee Moffitt Cancer Center & Research Institute, Tampa, Florida, United States of America; 2 Experimental Therapeutics Program, H. Lee Moffitt Cancer Center & Research Institute, Tampa, Florida, United States of America; 3 Analytic Microscopy Core, H. Lee Moffitt Cancer Center & Research Institute, Tampa, Florida, United States of America; 4 Department of Sarcoma Oncology, H. Lee Moffitt Cancer Center & Research Institute, Tampa, Florida, United States of America; 5 Department of Diagnostic Imaging, H. Lee Moffitt Cancer Center & Research Institute, Tampa, Florida, United States of America; NIH, United States of America

## Abstract

Combinations of targeted drugs have been employed to treat sarcomas, however, response rates have not improved notably, therefore emphasizing the need for novel treatments. In addition, imaging approaches to assess therapeutic response is lacking, as currently measurable indices, such as volume and/or diameter, do not accurately correlate with changes in tumor biology. In this study, quantitative and profound analyses of magnetic resonance imaging (MRI) were developed to evaluate these as imaging biomarkers for MK1775 and Gem in an osteosarcoma xenotransplant model at early time-points following treatment. Notably, we showed that Gem and Gem+MK1775 groups had significantly inhibited tumor growth by day 4, which was presaged by elevations in mean ADC by 24 hours post treatment. Significant differences were also observed at later time points for the Gem+MK1775 combination and MK1775 therapy. ADC distribution and entropy (randomness of ADC values) were also elevated by 24 hours following therapy. Immunohistochemistry demonstrated that these treatment-related increases in ADC correlated with apoptosis and observed cell condensations (dense- and exploded bodies). These findings underline the role of ADC as a quantitative imaging biomarker for therapy-induced response and show promising clinical relevance in the sarcoma patient population.

## Introduction

Sarcomas are malignancies of mesenchymal origin that develop within the supporting connective tissues of the body. Although rare in adults (i.e. 1% of all adult cancers), sarcomas are prevalent in children and young adolescents and account for 15% of all childhood cancers[1]. Sarcomas are classified as soft tissue cancers (myoleio, myxoid, Ewing's, lipo), gastrointestinal stromal tumors (GIST) and primary bone cancers (osteosarcomas), with the latter being the focus of this work.

Osteosarcoma, the most common type of malignant bone cancer, has a peak incidence in the second decade of life and affects more males than females[2], [3]. Osteosarcoma arises within bone and may, with time spread to the lungs, making the need for early detection and systemic chemotherapy treatment crucial to maximize survival. Similar to other cancers, the current mainstay of osteosarcoma therapy is surgical resection of the primary lesion and chemotherapy. The development of novel therapeutics for sarcomas is hampered by the low incidence and the inter- and intratumoral heterogeneity. Hence, although combinations of novel targeted drugs have been investigated and used, cure rates have only modestly improved in the last decades[4]. Thus, to improve survival and quality of life in sarcoma patients, there is a pressing need to develop novel therapies that exhibit high efficacy.

Previously, we have evaluated the effectiveness of a novel Wee-1 cell cycle checkpoint inhibitor, MK1775, in combination with gemcitabine for its potential to treat osteosarcomas [5]. In a subsequent study, we observed that MK1775 led to cell death in all sarcoma cells and tumor explants independent of their p53 status and showed strong synergistic interaction with gemcitabine[6]. Furthermore, we demonstrated that in a patient-derived osteosarcoma xenotransplant mouse model, MK1775 alone, and in combination with gemcitabine, caused marked terminal differentiation, apoptotic cell death and increased DNA damage by day 14. Because of the aforementioned challenges to measure response by traditional imaging modalities, we have investigated alternative methods that can predict clinical response to MK1775. In this current study, we investigated diffusion MRI at 7 T to evaluate the therapeutic effects of MK1775 and gemcitabine in patient-derived xenotransplant mouse models, particularly at early time-points.

MRI is the modality of choice for evaluating sarcomas and pre- and post gadolinium enhanced T_1_- and T_2_-weighted multiplanar imaging is currently the clinical standard-of-care (SOC). Due to its excellent tissue contrast and ability to provide anatomical detail in multiple planes, MRI is used in SOC to evaluate measures of response, such as size, necrosis and presence of contrast enhancement. However, it is difficult to correlate changes in these parameters with response to treatment and patient survival in sarcoma[7]. This is likely due to the inherent heterogeneous nature of these tumors. Furthermore, therapy-induced volume changes often occur later and cannot be reliably used as a measure of response during the early course of treatment. Therefore, the need for novel modalities that can assess functional and biologic information more accurately and at an earlier stage in the treatment is of utmost importance in order to affect patient care.

As such, more sophisticated MR approaches including dynamic contrast enhanced MRI (DCE-MRI)[8]–[10] and ^1^H magnetic resonance spectroscopy (MRS) [11], [12] have been used to predict therapeutic response in a variety of cancers, including sarcoma. In a recent study Guo et al showed that DCE-MRI could be an early imaging indicator of osteosarcoma treatment outcomes; *viz.* histologic response, event-free survival (EFS) and overall survival)[10]. An enduring challenge in these studies, though, is standardization and reproducibility of DCE-MRI[13], which generally requires an accurate choice of input function[14]. Nonetheless, this highlights the potential role of functional MR-modalities as biomarkers of response for osteosarcoma.

An appealing alternative MRI technique is diffusion-weighted MRI (DWI), which generally has a lower test-retest variance[15]. DWI provides information about the extent and direction of restricted water motion. If data are acquired with different diffusion-weightings (b-values), water mobility in biological tissue can be quantified to yield Apparent Diffusion Coefficients (ADC). It has been shown in numerous studies that the ADC is strongly affected by temperature, extra-cellular space as well as tortuosity, cellularity and integrity of cell membranes [8], [16], [17]. By quantifying ADC, DWI may therefore serve as a quantitative biomarker for determining lesion cellularity prior to treatment and subsequent changes in cellularity (cytotoxic or vasogenic edema) or loss of cell membrane integrity that may occur in response to successful therapy. Several previous studies have demonstrated increases in ADC values at early time-points following treatment correlated to cytotoxicity [18]–[20], while others have shown early decreases following treatment of anti-angiogenic therapy to reduce vasogenic edema [21], [22]. Responses of ADC to therapy in sarcoma have been inconsistent [23], [24] and we propose that such studies are limited by evaluating the entire tumor as a region of interest (ROI). This is especially true in sarcomas, which demonstrate significant intratumoral heterogeneity and thus variations in response to therapy and clinical behavior [25].

Thus, to establish a sensitive MRI-based approach for evaluating early chemotherapeutic response, we acquired T_2_-weighted Fast Spin-Echo (FSE) datasets for quantification of tumor volume and diffusion-weighted sequences (DW-FSE) to assess ADC properties within the tumors and potential therapy-induced alterations therein. To complement mean ADC calculations, we also quantified incremental ADC map pixel fractions, area under the difference (post response) ADC curves and the lack of symmetry in the ADC distribution (*i.e.* skewness and kurtosis) prior to and after, treatment. Describing the randomness in ADC values, ADC entropy also was determined to investigate potential therapy-induced differences in tumor texture. As a final corroboration of the *in vivo* MR data, histological staining with cleaved Caspase 3 (CC3) for apoptosis, γH2AX for DNA damage and standard Hematoxylin & Eosin (H&E) was performed and quantified for tissue obtained by day 4.

## Materials and Methods

### Experimental Setup

Animal procedures and xenotransplant protocols were approved by the Institutional Animal Care and Use Committee and Institutional Review Board (University of South Florida). As previously described, fresh tissue was obtained from a chemo-naïve, 52 year old osteosarcoma patient at the time of initial core biopsy of a distal femur osteosarcoma, which was metastatic to the lungs at the time of presentation[6]. The patient provided written informed consent. Patient tumor was implanted subcutaneously into both flanks of 16 six-week-old female SHO/SCID athymic mice (Charles River Laboratory). At a tumor volume of 500 mm^3^, mice were randomly assigned to the following treatment groups with N = 4 and thus 8 tumors per group: (1) control; (2) MK1775 (30 mg/kg. p.o. twice daily on days 1, 3, 8 and 10); (3) gemcitabine (100 mg/kg, i.p. once daily on days 1, 3, 8, 10); (4) MK1775+ gemcitabine (Combo) in the above mentioned doses. Drug doses and schedules were chosen based on the investigator brochure[6], [26], [27].

Prior to imaging, mice were anesthetized using 2% isoflurane and restrained in a mouse cradle. Respiratory function was monitored using the SAII system (Small Animal Instruments, Inc.) and temperature control was achieved by a gas unit, set to maintain a body temperature of 37±1°C.

### Imaging Protocol and Analysis

MRI was performed at baseline and 24 hours following each drug administration, and on day 14. All data were acquired using a 7-T horizontal magnet (ASR 310, Agilent Technologies, Inc.) equipped with nested 205/120/HDS gradient insert and a bore size of 310 mm. Using a 72-mm quadrature birdcage coil (Agilent Technologies, Inc.), axial T_2_-weighted fast spin-echo (FSE) sequences were acquired (TE/TR = 60/1403 ms) with slice thickness of 1.5 mm and in-plane resolution of 136 µm over 6 minutes. Applying identical slice plane and spatial resolution, diffusion weighted (DW) FSE sequences using four b-values  = 50, 500, 1000 and 2000 and TE/TR  = 36/1881 ms also were acquired over 12 minutes.

Image reconstruction and volumetric analyses were performed in VnmrJ (Agilent Technologies, Inc.) while ADC analysis used in-house routines in MATLAB. Manually drawn regions of interest (ROIs) encompassing the tumors were applied to the T_2_- and diffusion-weighted datasets for tumor volume and ADC. Tumor ADC was calculated using the following equation: S(b) = S(0)×e^−ADC*b^ where b is the diffusion sensitizing factor, S(b) and S(0) the signal intensity with and without diffusion weighting, respectively. Percentage change in mean ADC was calculated according to: ΔADC [%] = (ADC_t_/ADC_0_)×100, where ADC_0_ denotes the baseline ADC value. Similarly, the percent change in tumor volume was calculated as ΔV [%] = (V_t_/V_0_)×100 with V_0_ being the tumor volume obtained pre-treatment. ADC maps were generated by nonlinear least squares regression of a mono-exponential to the experimental signal intensity for all four b-values and.

To compare across ADC values in a given tumor, an incremental lower ADC limit was set at 3×10^−5^ mm^2^/s, and by 512 increments, all values above the lower threshold with an upper limit of 5×10^−3^ were quantified and plotted to compare pre- and post treatment for all time points and mice[28]. Therapy-induced alterations in ADC values were further characterized by area under the curve (AUC) analysis, which was performed by subtracting the pre-treatment ADC pixel fraction curve from the post-treatment curve for each animal. Changes in skewness and kurtosis also were quantified and are displayed in histograms showing the difference between baseline values pre-treatment and 24 hours following treatment.

### Texture Analysis

Visual analysis demonstrated intra-tumoral change and heterogeneity in the diffusion-weighted datasets, which appeared to correlate with treatment. To quantify these variations and subtract image texture features (i.e. distinct regions or repeating patterns of pixelated behavior), entropy filtering was applied to the ADC maps [29] [30]. Entropy filter is the implementation of Shannon entropy; a texture-based statistical measure of the variation in the histogram distribution, which reflects the predictability of intensity values within a given ROI. The region was manually delineated to encompass the entire tumor and a set of consecutive masks was generated, thus forming a volume of interest (VOI).

The entropy of the ADC maps was computed using the values within ADC map VOI. A custom entropy algorithm was implemented in MATLAB. Due to size limitations of the ADC maps (128×128 pixels per horizontal slice), entropy computation was performed globally over the entire VOI and the ADC values were used to form a histogram vector, which was subdivided into four discrete bins. For each tumor, a single entropy coefficient was computed and used as a measure of variability within that VOI.

Intensity variations within individual VOIs were visualized using 3D graphs of entropy coefficients. Instead of computing one entropy score for the entire tumor, entropy was computed for each pixel based on the ADC values of its neighboring pixels (9×9 window), which were also within the tumor VOI. Each pixel was then color-coded based on the value of its local entropy coefficient.

### Histological Evaluation

Employing the identical experimental setup, animal experiments were performed in two sets of mice. The first batch (N = 16), focused on the cytotoxic effects of MK-1775 and Gem in osteosarcoma xenografts through day 14, with this as the immunohistochemical endpoint [6]. Quantitative analysis of these datasets demonstrated significant ADC increases by 2 to 4 days following therapy. Therefore, the current study (N = 16) focused on these early times-points with immunohistochemistry performed on day 4. Histological processing and staining was performed as previously described[6].

H&E stained histology slides of xenograft tumors, γH2AX and CC3 were scanned using the Aperio™ (Vista, CA) ScanScope XT with a 200×/0.8NA objective lens at a rate of 5 minutes per slide via Basler tri-linear-array. Histological pattern recognition was conducted using the Genie® (Aperio, Vista, CA, USA) software platform to segment and classify necrosis, viable tumor and other non-target tissues (i.e. adipose, muscle and skin). In addition, TissueStudio® (Definiens, Munich, Germany) image analysis platform was used to segment, classify and extract size and intensity features for single cells in the viable tumor and necrotic regions of each image.

Quantification of CC3 stained exploded bodies was performed by spectrally unmixing images into hematoxylin and DAB channels using the TRIO image acquisition and analysis system (Caliper; Boston, MA). Each channel was rerun through the segmentation and classification algorithm described earlier and the number of exploded bodies, with, and without CC3 staining was recorded.

### Statistical Analysis

Statistical analysis was performed using Graphpad Prism software (Graphpad, San Diego, CA, USA) and one-way analysis of variance (ANOVA) followed by the Tukey test for comparison of mean values. A confidence interval of 95% was chosen and thus statistical significance was pre-determined at p<0.05. For each measured parameter (i.e. tumor volume, mean ADC values, ADC-AUC, entropy and histological scoring), the averaged mean value for each of the four cohorts on a specific day was compared to one another and the results are presented in Table 1.

**Table 1:** P-values obtained from a one-way analysis of variance (ANOVA) followed by the Tukey test for comparison of mean values between the stated groups on the specified time points for (A) tumor volumes, (B) percent change in mean ADC, (C) ADC-AUC analysis and (D) CC3 and γH2AX staining.

*A: Day*	C vs M	C vs G	C vs X	M vs G	M vs X	G vs X
2	0.71	0.30	0.23	0.94	0.88	1.00
4	0.10	0.005*	0.001*	0.024*	0.004*	0.86
9	0.99	0.22	0.24	0.45	0.52	0.99
11	>1.00	-	0.20	-	0.34	-
14	-	0.19	0.025*	-	-	0.79

*B: Day*	C vs M	C vs G	C vs X	M vs G	M vs X	G vs X
2	0.38	0.005*	0.007*	0.36	0.35	1.00
4	1.00	0.09	0.23	0.09	0.23	0.92
9	0.20	0.12	<0.001*	0.83	0.037*	0.53
11	0.80	-	0.27	-	0.89	-
14	-	0.74	>1.00	-	-	0.76

*C: Day*	C vs M	C vs G	C vs X	M vs G	M vs X	G vs X
2	0.53	0.31	0.039*	0.98	0.53	0.73
4	0.99	0.035*	0.43	0.11	0.70	0.51
9	0.26	0.43	0.004*	1.00	0.56	0.62
11	0.41	-	0.18	-	0.98	-
14	-	0.47	0.99	-	-	0.43

*D: Day 4*	C vs M	C vs G	C vs X	M vs G	M vs X	G vs X
CC3	0.95	<0.001*	0.005*	0.001*	0.020*	0.82
γH2AX	>1.00	0.29	0.19	0.34	0.24	0.99

% was chosen and statistical significance determined at p<0.05. A confidence interval of 95

*indicates significant differences between the stated groups which were abbreviated as follows; C =  controls, M =  MK1775, G =  gemcitabine and X =  combination group. Each group was composed of 4 mice with two flank tumors each, thus N = 8 per group.

## Results

### High Resolution MR datasets and Volumetric Analysis

**Figure 1:**
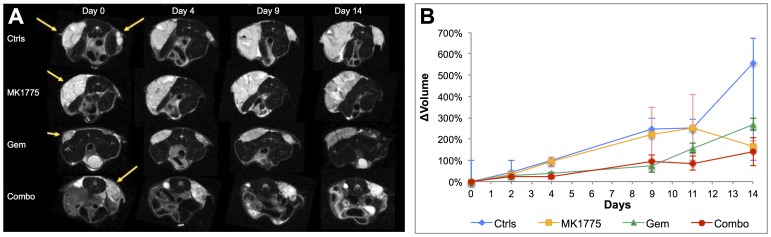
Longitudinal monitoring of tumor growth using MRI. (**A**) T_2_-weighted fast spin echo datasets (TE/TR = 16/1403 ms) at 136 µm resolution demonstrating the visual difference in tumor growth pre-treatment and on days 4, 9 and 15 for all groups. (**B**) Percent relative change in average tumor volumes from day 0 showing significantly larger tumors for the controls and MK1775 treated animals compared to the Gem and Combo groups on day 4. Treatments were administered on days 1, 3, 8 and 10.

Figure 1A shows that high resolution FSE datasets allow for the delineation of the osteosarcoma tumors for all animals at all imaging time points. The borders of each tumor were distinctly visible making the lesions easy to distinguish from surrounding tissue and thereby facilitating segmentation for volume and diffusion properties. In addition, these images also displayed heterogeneity and microstructural details within the tumors, particularly with an increase in tumor size. Figure 1A further displays the difference in tumor sizes between the various groups. Figure 1B shows the time course of tumor volume quantifications, which showed that the Gem and Combo groups appeared to exhibit decreased tumor growth relative to the MK1775 and control tumors. Both Gem and Combo groups had significantly lower tumor volumes on day 4, but by day 14 only the Combo group was significantly lower than the untreated controls (Table 1A). The MK1775 group grew similar to the controls up until day 11, but appear to have decreased tumor volumes by day 14. However, this can be explained by having only 2 remaining quantifiable imaging acquisitions from this group on day 14 and this possible decrease cannot be statistically verified.

### Quantification of ADC, Skewness and Kurtosis

**Figure 2:**
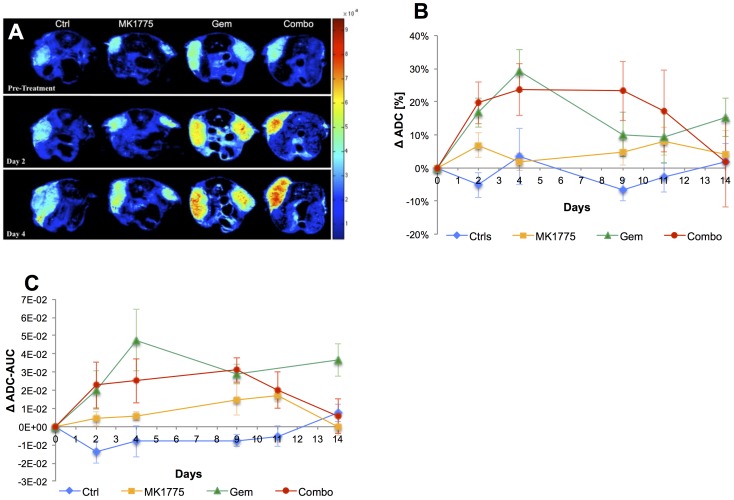
Assessment of diffusion properties and potential alterations between treatment groups following therapy. (**A**) Parametric ADC maps demonstrating restricted tumor diffusivity for all four groups comparing pre-treatment to day 2 and 4 post-therapy. (**B**) Quantitative analysis of mean ADC plotted as percent change from baseline (pre-treatment) over the experimental time-line showing statistical significance between groups already by day 2 (24 hours following the first treatment on day 1). (**C**) To compare across mice, ADC-AUC for each mouse was quantified by subtracting pre-treatment ADC pixel fraction curves from post-therapy and plotted as the average value for each group at all time points.

To investigate the response of apparent water diffusion across the tumors to therapy, ADC maps were generated for all animals. Following established protocols, animals were treated on days 1, 3, 8 and 10. Focusing on the early time points, Figure 2A demonstrates a notable increase in ADC by 24 hours following the first and second treatment (i.e. day 2 and 4, respectively) in the Gem and Combo groups while the controls and MK1775 showed that the ADC was unaltered. To quantify this effect, each animal was used as its own control, and the mean ADC was estimated, then group averaged and displayed as percent change from the baseline value with standard deviations. As shown in Figure 2B, the ADC of the control group and MK1775 did not change significantly over the course of the study. The Gem group had a dramatic and significant increase in ADC evident by day 2, which persisted until day 9, and increased slightly following the last dose on day 10. Similar to the Gem cohort, the combo group demonstrated a significant increase in ADC following initiation of therapy, but in contrast to Gem, the ADC remained elevated until 5 days following the last treatment, at which time these tumors had started to re-grow (cf. Figure 1B). Statistically, significant differences in ADC were established for the controls compared to Gem and Combo on day 2, as well as controls and Combo, and MK1775 and Combo, on day 9 (Table 1B).

ADC histograms were generated for each mouse prior to and following therapy and using a similar approach as Galons *et al*, these were used to generate cumulative pixel fraction plots[28]. Subtracting the pre- from the post-therapy pixel fraction plots is a sensitive measure of movement to higher ADC values and the area under this resulting curve (AUC) was calculated and plotted as the average value for each treatment group as a function of time (Figure 2C). These results were in agreement with both volumetric and mean ADC analysis and demonstrated significantly larger shifts in AUC for the Gem and Combo groups than MK1775 and controls at most time points (Table 1C). Statistical significance was reached between control and Combo on day 2 and 9, and control and Gem on day 4.

**Figure 3:**
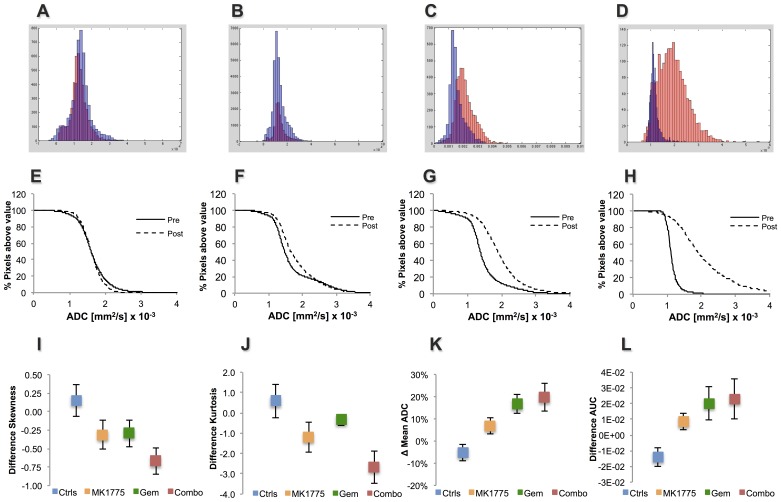
Analysis of ADC distribution within tumors prior to- and following therapy. (**A–D**) Pixel-by-pixel histograms demonstrating the ADC distribution pre- (blue) and post-treatment on day 2 (red) for select animals from all four treatment groups. (**E–H**) Corresponding incremental ADC pixel fraction plots for representative animals showing a shift of ADCs towards higher values in Gem and Combo treated animals compared to MK1775 and control. Quantitative analysis comparing pre-treatment to day 2 of (**K**) relative skewness and (**L**) kurtosis and comparison with (**I**) percent change in mean ADC, (**J**) AUC values.

To further characterize diffusion properties within the tumors, additional descriptive statistical measures of the ADC distribution; *viz.* skewness and kurtosis, were determined. Skewness and kurtosis describe the lack of symmetry and the breadth of the ADC pixel distribution, respectively, and alterations therein are direct indicators of ADC changes across tumors. Figures 3A–D show the raw ADC histograms of the pixel distributions pre- and 24 hours post the first treatment. Whereas the histogram for a representative control animal showed similar ADC pixel distributions prior to and following treatment, it is evident that the treated animals showed alterations within 24 hours after the first drug administration. In particular, the ADC distribution post-treatment appears to be markedly skewed and have a broader peak for the Combo, and to a lesser extent the Gem and MK1775 groups. The ADC histograms thus correlate to the incremental ADC pixel fraction plots, which demonstrate a shift of ADCs towards higher values in Gem and Combo treated animals compared to MK1775 and controls (Figures 3 E–H).

Quantitatively, the relative difference in skewness and kurtosis for each group 24 hours following treatment is shown in Figures 3I and 3J. In agreement with the mean ADC and AUC data at the same time point (Figure 3K & L), all three treated groups, and Combo in particular, demonstrated negative skewness and kurtosis following therapy while the control group showed a minor positive increase. Although this negative trend was evident for all three treated groups, statistical significance could only be established between controls and Combo (p = 0.018 and p = 0.043, skewness and kurtosis respectively) at 24 hours.

### Tumor Microstructure and Entropy

**Figure 4:**
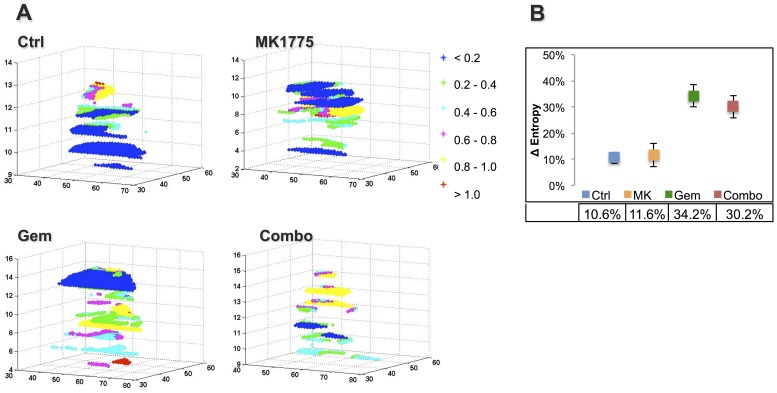
The randomness of ADC values within tumors pre- and post therapy were visualized and quantified using Shannon Entropy in Matlab. (**A**) ADC-entropy plots as obtained by texture-based analysis of post-treatment ADC-maps herein demonstrating the evident differences in ADC values and distribution within the perimeters of the tumor (see color bar). The axes represent spatial dimensions (*i.e.* pixel coordinates). (**B**) Corresponding change in average entropy values from pre-treatment for all four groups displaying statistically larger variations in ADC values for the Gem group compared to both controls (p = 0.022) and MK1775 (p = 0.023).

Entropy is a texture-based statistical measure of the randomness in an ADC histogram such that a large variation in ADC is associated with higher entropy values. Presented in Figure 4A, post treatment ADC entropy plots on day 2 demonstrate evident differences between the various groups. The most apparent distinction is between the controls and the Combo and Gem groups, with the controls showing large regions of substantially lower entropy values (blue color). In addition, the entropy distributions varied considerably for all treated groups, and especially so in Gem and Combo in particular, compared to controls. In agreement with this visual assessment, the quantitative data demonstrated in Figure 4B shows a significantly larger change in ADC entropy with treatment for the Gem (34.2%) group compared to both MK1775 (11.6%, p = 0.023) and controls (10.6%, p = 0.022). While the Combo group also displayed a 30.2% increase in ADC-entropy compared to pre-treatment values, statistical significance could not be established.

### Quantitative Immunohistochemistry (CC3, γH2AX and H&E)

**Figure 5:**
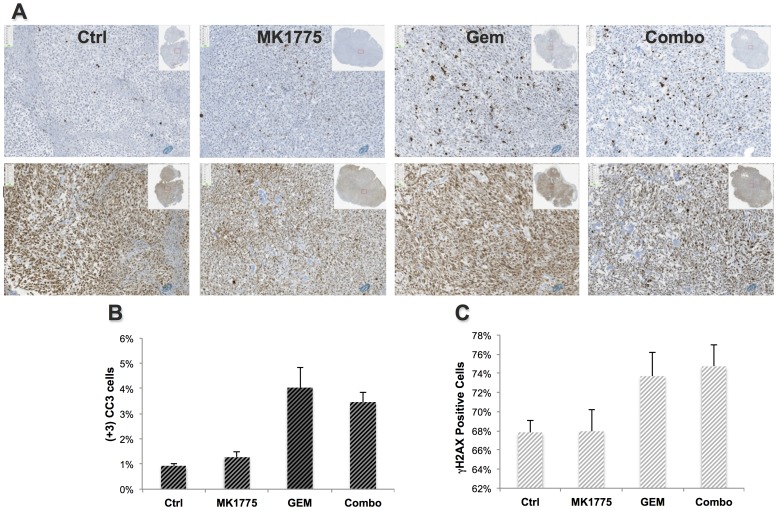
Quantified histological staining demonstrating the difference in apoptotic (CC3) burden and DNA damage between groups. (**A**) Microscopic images (10X) of CC3 (row 1) and γH2AX (row 2) stained sections of representative mice from each group on day 4 demonstrating higher apoptosis and DNA-damage in the Gem and Combo groups and corresponding quantitative analysis of percent (**B**) apoptosis and (**C**) DNA damage.

For the evaluation of drug-mediated cytotoxic and apoptotic effects, immunohistochemical analyses were performed with cleaved caspase 3 (CC3) antibody and phosphorylated histone (γH2AX). As a marker for apoptotic cell death, CC3 activity was significantly higher on day 4 in the Gem and Combo groups compared to controls but were not elevated in the MK1775 group (Figure 5A and B, Table 1D). The γH2AX staining appears elevated in the GEM and Combo groups (Figure 5C), however these differences are not statistically significant.

**Figure 6:**
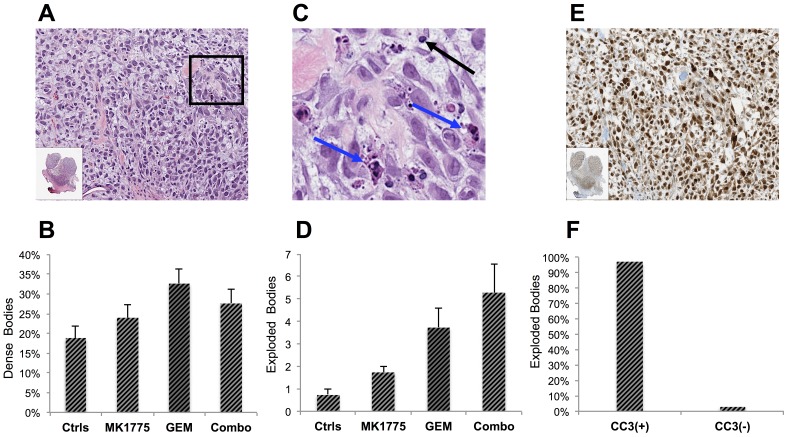
Quantitative analysis of unusually dense and “exploded” masses demonstrating a correlation with treatment response. (**A**) A standard H&E section of a Gem-treated tumor on day 4 imaged with 20X zoom and (**B**) quantitative analysis showing percent dense bodies in comparison with normal cells. (**C**) Insert demonstrates a higher burden of unusually small and dense cells (black arrows) and large bulgy masses herein denoted as “exploded cells” (blue arrows). (**D**) Amount exploded bodies per tumor section indicating an increase with treatment response. (**E**) A CC3 stained tumor section demonstrating the amount of apoptosis as indicated by positively stained cells (brown) and (**F**) quantitative analysis of percent “exploded dense bodies” that stain fully for CC3 and are thus apoptotic.

Standard H&E sections also were analyzed, however, no visual or quantitative differences with regards to potential bone formation, necrosis or average cell area were detected at day 4 (Figure S1). Similarly, quantitative analysis of cellularity did not statistically differ between groups at this time-point, however, a trend was observed correlating treatment response with decreased cellularity (Figure S1B). Interestingly, as determined by microscopic analysis of the H&E stained sections, we noted a varying amount of small dark bodies that seemed to be particularly increased in the Gem and Combo groups (Figure 6A). Verifying these observations, a quantification of these dense units demonstrated a higher percentage in all treated groups, but Gem in particular (p = 0.038), compared to controls (Figure 6B). To further evaluate these unknown structures, the fractions of these bodies that stained positively for CC3 were determined. Although all three treated groups contained more apoptotic dense bodies than the controls, statistical significance could not be established between any of the groups.

We also observed dark dense bodies with exceedingly large and bulgy masses that were increased in the Gem and Combo groups compared to controls (Figure 6C). Classified as “exploded dense bodies”, quantification of these structures demonstrated significantly higher levels in the Gem (p = 0.048) and Combo (p = 0.004), than in controls (Figure 6D). The Combo group also showed significantly higher burden of these exploded units compared to the MK1775 treated mice (p = 0.032). To evaluate whether these exploded bodies stained positively for CC3 and thus comprised the apoptotic portion of these dense units demonstrated in Figure 6C, spectral unmixing image analysis was used to deconvolve and register the two signals and determined that 97% of these structures were fully apoptotic (Figure 6E & F). No intra-group differences were detected.

## Discussion

In this study, we used diffusion MRI to evaluate the response of osteosarcoma to Gem and MK1775. However, the focus of the current study was to evaluate quantitative approaches to assess biologically relevant therapy-induced responses at earlier time-points than conventional volumetric analysis. Specifically, we investigated multiple descriptors of ADC parameters and compared these to subsequent changes in tumor volumes and tumor microstructure.

Significant effects of therapy on tumor volume were observed by day 4 (72 hours after first dose) and the volume changes correlated with ADC. Significant increases in mean tumor ADC and ADC distribution could be detected within 24 hours following initiation of treatment, and these presaged subsequent volume responses. The Combo group had elevated ADC and tumor volume responses that were sustained at later time points relative to the GEM treatment alone. Quantitatively, these observations are in agreement with previous findings wherein ADC increased shortly after initiation of therapy in both humans and animal models [8], [17], [20], [31]–[33]. These rapid increases in ADC following treatment have been ascribed to reduced cellularity associated with cytotoxicity, as well as impaired cell membrane integrity associated with necrosis, both of which allow for more unimpeded diffusion [31], [34], [35]. Another contributing factor to elevated ADC values may be cell shrinkage due to increased interstitial osmolality or apoptosis [36], [37]. It should be noted, however, that some therapies can result in a decrease in ADC, through reduction of vascular permeability by anti-VEGF therapies, which leads to reduced vasogenic edema and reduced interstitial osmolality [38]. Hence, interpretation of ADC data requires accurate knowledge of the biology of the tumor and the anti-tumor therapy mechanism of action. Regardless of mechanism, increased ADC is interpreted to be synonymous with increased interstitial space, and vice-versa.

In this study, histological analysis did not demonstrate significant therapy-induced necrosis by day 4, however, a trend of reduced cellularity as well as a larger range in cell sizes for the treated groups was observed, which may be explained by the presence of the unusually smaller dense and exploded bodies. More important, these findings correlated with the increases in mean ADC. For all animals, this increase in ADC also was associated with a negatively skewed ADC distribution as represented by the skewness and kurtosis. Consistently, the Combo group had more exploded bodies and greater perturbations in skewness and kurtosis of the ADC histogram compared to GEM or MK1775 alone.

Skewness and kurtosis are statistical measures of the asymmetry and breadth of a distribution, respectively. Theoretically, if the entire distribution moves to higher values, the mean ADC should yield the greatest statistically significant changes, as was the case in this current study. Previously, assessments of ADC skewness and kurtosis have been applied for various tumors; however, its usefulness thus far has been limited to the brain [39]–[41]. Capturing a different microenvironment for diffusivity, ADC histograms provide information that may be masked by mean ADC values [20], and should therefore complement standard ADC analysis. Further investigating ADC distribution, we found incremental ADC pixel fraction plots and ADC-AUC analysis informative in establishing therapy response. Providing quantitative information about ADC distribution, AUC analysis comparing pre- and post-treatment plots, showed statistically significant differences between groups, which also correlated with volume, mean ADC, skewness and kurtosis.

Perhaps more intriguing, ADC-entropy measurements and 3-dimensional entropy plots demonstrated the intra-tumoral ADC variability and correlated with tumor growth, mean ADC, ADC distribution, AUC analysis and histology. Describing the variability of ADC values within a certain ROI, ADC entropy calculations were first reported in the literature by Benedict *et al* in 2007 and was therein recognized as a sensitive marker of overall brain damage that strongly correlated with cognitive impairment [29]. More recently, a study by Kierans *et al*. demonstrated that ADC entropy showed significantly greater accuracy than mean ADC for characterization of malignancy of adnexal lesions [30]. In this current study, ADC entropy was able to predict treatment response following cancer therapy at earlier time-points than tumor volume changes. To our knowledge, this application of ADC-entropy has previously not been investigated nor reported.

The final segment of our approach evaluated potential mechanisms responsible for these observed therapy-induced changes in ADC and ADC distribution. On day 4 following therapy, we found that CC3 staining was significantly elevated in the Gem and Combo groups relative to controls and MK1775, indicating that apoptosis was a mechanism of rapid treatment response. This correlates well with the peak increases in ADC that occurred on day 4 for these two groups.

Equally compelling, was a significant increase in condensed cell bodies in the Gem group relative to controls. A subset of these dense bodies had an “exploded” appearance, and were highly correlated with all three treatment groups. As determined by quantitative image analysis, 98% percent of these dense exploded bodies stained fully for CC3 and, thus, apoptosis most likely is the cell-death mechanism leading to the formation of these condensed bodies. Since the presence of CC3 is a temporal event in the pathway to apoptotic cell death, the inability to detect CC3 in all dense bodies does not rule out apoptosis as being the sole mechanism. However, it is possible that apoptosis occurs in only a subset of cells undergoing cell condensation.

Interestingly, while MK1775 did not induce significant apoptosis or elevated ADC values at day 4, our studies have shown that when administered for a prolonged time period (i.e. 10 days or longer), there is a cumulative therapeutic effect of MK1775 as a mono-therapy. In addition, histological differences indicating differentiation (osteogenesis) and DNA damage are clearly evident by day 14. Therefore, our results suggest that ADC is a predictive biomarker for acute cytotoxic effects, however, not as sensitive to predict slower, cumulative processes that occur at later time points.

In conclusion, therapeutic effects of Gem, administered solely or in combination with MK1775, induced significant tumor growth control by day 4, which was presaged by significant elevations in mean tumor ADC, ADC distribution and entropy by 24 hours following therapy. Treatment related increases in ADC, CC3 staining and observed cell condensations indicated that apoptosis was a significant component of the response to treatment regimens that included Gem and that the increased ADC values in this study correlated with the apoptotic response. At early time points, the combination GEM+MK1775 showed significantly increased responses relative to GEM or MK1775 alone by multiple measures. As expected, if tumor control occurs more slowly, e.g. with MK1775 monotherapy, there are no significant changes in ADC or ADC distribution, although there is a slight trend to higher values. We interpret this observation to indicate that therapy-induced edema can be systemically absorbed faster than it is created. These results show promising clinical relevance in the sarcoma patient population as agents that can induce a rapid response can be quantitatively assessed at an earlier stage in the treatment, thereby impacting clinical management.

## Supporting Information

Figure S1 Visual and quantitative analysis of standard H&E sections herein displayed with 10X zoom. (**A**) H&E sections from each group were analyzed for potential bone formation, necrosis, number of nuclei and average cell area. (**B**) Cell number analysis across groups indicates a trend of fewer cells in the treated groups compared to controls and. (**C**) Quantification of average cell area for each tumor and group also indicates a larger range in cell sizes for the treated groups.
(TIFF)

## References

[1] Joseph L, Trent JC (2008) Targeted Cancer Therapy: Humana Press.

[2] Marulanda GA, Henderson ER, Johnson DA, Letson GD, Cheong D (2008) Orthopedic surgery options for the treatment of primary osteosarcoma. Cancer Control 15: 13–20. 18094657 10.1177/107327480801500103

[3] Vander Griend RA (1996) Osteosarcoma and its variants. Orthop Clin North Am 27: 575–581. 8649738

[4] Blanke CD, Rankin C, Demetri GD, Ryan CW, von Mehren M, et al (2008) Phase III randomized, intergroup trial assessing imatinib mesylate at two dose levels in patients with unresectable or metastatic gastrointestinal stromal tumors expressing the kit receptor tyrosine kinase: S0033. J Clin Oncol 26: 626–632. 18235122 10.1200/JCO.2007.13.4452

[5] Kreahling JM, Gemmer JY, Reed D, Letson D, Bui M, et al (2012) MK1775, a selective Wee1 inhibitor, shows single-agent antitumor activity against sarcoma cells. Mol Cancer Ther 11: 174–182. 22084170 10.1158/1535-7163.MCT-11-0529 PMC4545500

[6] Kreahling JM, Foroutan P, Reed D, Martinez G, Razabdouski T, et al (2013) Wee1 inhibition by MK-1775 leads to tumor inhibition and enhances efficacy of gemcitabine in human sarcomas. PLoS One 8: e57523. 23520471 10.1371/journal.pone.0057523 PMC3592874

[7] Stacchiotti S, Collini P, Messina A, Morosi C, Barisella M, et al (2009) High-grade soft-tissue sarcomas: tumor response assessment–pilot study to assess the correlation between radiologic and pathologic response by using RECIST and Choi criteria. Radiology 251: 447–456. 19261927 10.1148/radiol.2512081403

[8] Jordan BF, Runquist M, Raghunand N, Baker A, Williams R, et al (2005) Dynamic contrast-enhanced and diffusion MRI show rapid and dramatic changes in tumor microenvironment in response to inhibition of HIF-1alpha using PX-478. Neoplasia 7: 475–485. 15967100 10.1593/neo.04628 PMC1501160

[9] Cárdenas-Rodríguez J, Li Y, Galons JP, Cornnell H, Gillies RJ, et al (2012) Imaging biomarkers to monitor response to the hypoxia-activated prodrug TH-302 in the MiaPaCa2 flank xenograft model. Magn Reson Imaging 30: 1002–1009. 22554971 10.1016/j.mri.2012.02.015 PMC3402593

[10] Guo J, Reddick WE, Glass JO, Ji Q, Billups CA, et al (2012) Dynamic contrast-enhanced magnetic resonance imaging as a prognostic factor in predicting event-free and overall survival in pediatric patients with osteosarcoma. Cancer 118: 3776–3785. 22180392 10.1002/cncr.26701 PMC3310962

[11] Hakumäki JM, Poptani H, Puumalainen AM, Loimas S, Paljärvi LA, et al (1998) Quantitative 1H nuclear magnetic resonance diffusion spectroscopy of BT4C rat glioma during thymidine kinase-mediated gene therapy in vivo: identification of apoptotic response. Cancer Res 58: 3791–3799. 9731486

[12] Abdel Razek AA, Poptani H (2013) MR spectrsocopy of head and neck cancer. Eur J Radiol 82: 982–989. 23485098 10.1016/j.ejrad.2013.01.025

[13] Padhani AR, Leach MO (2005) Antivascular cancer treatments: functional assessments by dynamic contrast-enhanced magnetic resonance imaging. Abdom Imaging 30: 324–341. 15688112 10.1007/s00261-004-0265-5

[14] Zhang X, Lin Y, Gillies RJ (2010) Tumor pH and its measurement. J Nucl Med 51: 1167–1170. 20660380 10.2967/jnumed.109.068981 PMC4351768

[15] Jordan BF, Gallez B (2010) Surrogate MR markers of response to chemo- or radiotherapy in association with co-treatments: a retrospective analysis of multi-modal studies. Contrast Media Mol Imaging 5: 323–332. 20648644 10.1002/cmmi.397

[16] Patterson DM, Padhani AR, Collins DJ (2008) Technology insight: water diffusion MRI–a potential new biomarker of response to cancer therapy. Nat Clin Pract Oncol 5: 220–233. 18301415 10.1038/ncponc1073

[17] Jennings D, Hatton BN, Guo J, Galons JP, Trouard TP, et al (2002) Early response of prostate carcinoma xenografts to docetaxel chemotherapy monitored with diffusion MRI. Neoplasia 4: 255–262. 11988845 10.1038/sj.neo.7900225 PMC1531699

[18] Fujimoto H, Kazama T, Nagashima T, Sakakibara M, Suzuki TH, et al.. (2013) Diffusion-weighted imaging reflects pathological therapeutic response and relapse in breast cancer. Breast Cancer. 10.1007/s12282-013-0449-3 23400545

[19] Uhl M, Saueressig U, Koehler G, Kontny U, Niemeyer C, et al (2006) Evaluation of tumour necrosis during chemotherapy with diffusion-weighted MR imaging: preliminary results in osteosarcomas. Pediatr Radiol 36: 1306–1311. 17031633 10.1007/s00247-006-0324-x

[20] Kyriazi S, Collins DJ, Messiou C, Pennert K, Davidson RL, et al (2011) Metastatic ovarian and primary peritoneal cancer: assessing chemotherapy response with diffusion-weighted MR imaging–value of histogram analysis of apparent diffusion coefficients. Radiology 261: 182–192. 21828186 10.1148/radiol.11110577

[21] Farrar CT, Kamoun WS, Ley CD, Kim YR, Catana C, et al (2011) Sensitivity of MRI tumor biomarkers to VEGFR inhibitor therapy in an orthotopic mouse glioma model. PLoS One 6: e17228. 21390238 10.1371/journal.pone.0017228 PMC3048404

[22] Gerstner ER, Chen PJ, Wen PY, Jain RK, Batchelor TT, et al (2010) Infiltrative patterns of glioblastoma spread detected via diffusion MRI after treatment with cediranib. Neuro Oncol 12: 466–472. 20406897 10.1093/neuonc/nop051 PMC2940624

[23] Bajpai J, Gamnagatti S, Kumar R, Sreenivas V, Sharma MC, et al (2011) Role of MRI in osteosarcoma for evaluation and prediction of chemotherapy response: correlation with histological necrosis. Pediatr Radiol 41: 441–450. 20978754 10.1007/s00247-010-1876-3

[24] Oka K, Yakushiji T, Sato H, Hirai T, Yamashita Y, et al (2010) The value of diffusion-weighted imaging for monitoring the chemotherapeutic response of osteosarcoma: a comparison between average apparent diffusion coefficient and minimum apparent diffusion coefficient. Skeletal Radiol 39: 141–146. 19924412 10.1007/s00256-009-0830-7

[25] Skubitz KM, Pambuccian S, Manivel JC, Skubitz AP (2008) Identification of heterogeneity among soft tissue sarcomas by gene expression profiles from different tumors. J Transl Med 6: 23. 18460215 10.1186/1479-5876-6-23 PMC2412854

[26] Rajeshkumar NV, De Oliveira E, Ottenhof N, Watters J, Brooks D, et al (2011) MK-1775, a potent Wee1 inhibitor, synergizes with gemcitabine to achieve tumor regressions, selectively in p53-deficient pancreatic cancer xenografts. Clin Cancer Res 17: 2799–2806. 21389100 10.1158/1078-0432.CCR-10-2580 PMC3307341

[27] Leijen S, Beijnen JH, Schellens JH (2010) Abrogation of the G2 checkpoint by inhibition of Wee-1 kinase results in sensitization of p53-deficient tumor cells to DNA-damaging agents. Curr Clin Pharmacol 5: 186–191. 20406171 10.2174/157488410791498824

[28] Galons JP, Morse DL, Jennings DR, Gillies RJ (2003) Diffusion-Weighted MRI and Response to Anti-Cancer Therapies. Israel Journal of Chemistry. 91–101.

[29] Benedict RH, Bruce J, Dwyer MG, Weinstock-Guttman B, Tjoa C, et al (2007) Diffusion-weighted imaging predicts cognitive impairment in multiple sclerosis. Mult Scler 13: 722–730. 17613599 10.1177/1352458507075592

[30] Kierans AS, Bennett GL, Mussi TC, Babb JS, Rusinek H, et al (2013) Characterization of malignancy of adnexal lesions using ADC entropy: comparison with mean ADC and qualitative DWI assessment. J Magn Reson Imaging 37: 164–171. 23188749 10.1002/jmri.23794

[31] Morse DL, Galons JP, Payne CM, Jennings DL, Day S, et al (2007) MRI-measured water mobility increases in response to chemotherapy via multiple cell-death mechanisms. NMR Biomed 20: 602–614. 17265424 10.1002/nbm.1127

[32] Galons JP, Altbach MI, Paine-Murrieta GD, Taylor CW, Gillies RJ (1999) Early increases in breast tumor xenograft water mobility in response to paclitaxel therapy detected by non-invasive diffusion magnetic resonance imaging. Neoplasia 1: 113–117. 10933044 10.1038/sj.neo.7900009 PMC1508128

[33] Theilmann RJ, Borders R, Trouard TP, Xia G, Outwater E, et al (2004) Changes in water mobility measured by diffusion MRI predict response of metastatic breast cancer to chemotherapy. Neoplasia 6: 831–837. 15720810 10.1593/neo.03343 PMC1531687

[34] Moffat BA, Hall DE, Stojanovska J, McConville PJ, Moody JB, et al (2004) Diffusion imaging for evaluation of tumor therapies in preclinical animal models. MAGMA 17: 249–259. 15580371 10.1007/s10334-004-0079-z

[35] Kauppinen RA (2002) Monitoring cytotoxic tumour treatment response by diffusion magnetic resonance imaging and proton spectroscopy. NMR Biomed 15: 6–17. 11840548 10.1002/nbm.742

[36] Ackerman JH, Neil JJ (2010) Biophysics of Diffusion in Cells. Diffusion MRI. 1 ed. 110–124.

[37] Grant SC, Buckley DL, Gibbs S, Webb AG, Blackband SJ (2001) MR microscopy of multicomponent diffusion in single neurons. Magn Reson Med 46: 1107–1112. 11746576 10.1002/mrm.1306

[38] Seierstad T, Folkvord S, Røe K, Flatmark K, Skretting A, et al (2007) Early changes in apparent diffusion coefficient predict the quantitative antitumoral activity of capecitabine, oxaliplatin, and irradiation in HT29 xenografts in athymic nude mice. Neoplasia 9: 392–400. 17534444 10.1593/neo.07154 PMC1877980

[39] Pope WB, Kim HJ, Huo J, Alger J, Brown MS, et al (2009) Recurrent glioblastoma multiforme: ADC histogram analysis predicts response to bevacizumab treatment. Radiology 252: 182–189. 19561256 10.1148/radiol.2521081534

[40] Tozer DJ, Jäger HR, Danchaivijitr N, Benton CE, Tofts PS, et al (2007) Apparent diffusion coefficient histograms may predict low-grade glioma subtype. NMR Biomed 20: 49–57. 16986106 10.1002/nbm.1091

[41] Nowosielski M, Recheis W, Goebel G, Güler O, Tinkhauser G, et al (2011) ADC histograms predict response to anti-angiogenic therapy in patients with recurrent high-grade glioma. Neuroradiology 53: 291–302. 21125399 10.1007/s00234-010-0808-0 PMC3063200

