# Supplementary figures and images for: Diffusion MRI and Novel Texture Analysis in Osteosarcoma Xenotransplants Predicts Response to Anti-Checkpoint Therapy
Source: PLoS One. 2013 Dec 16;8(12):e82875. doi: 10.1371/journal.pone.0082875 (PMC3865096; doi:10.1371/journal.pone.0082875)

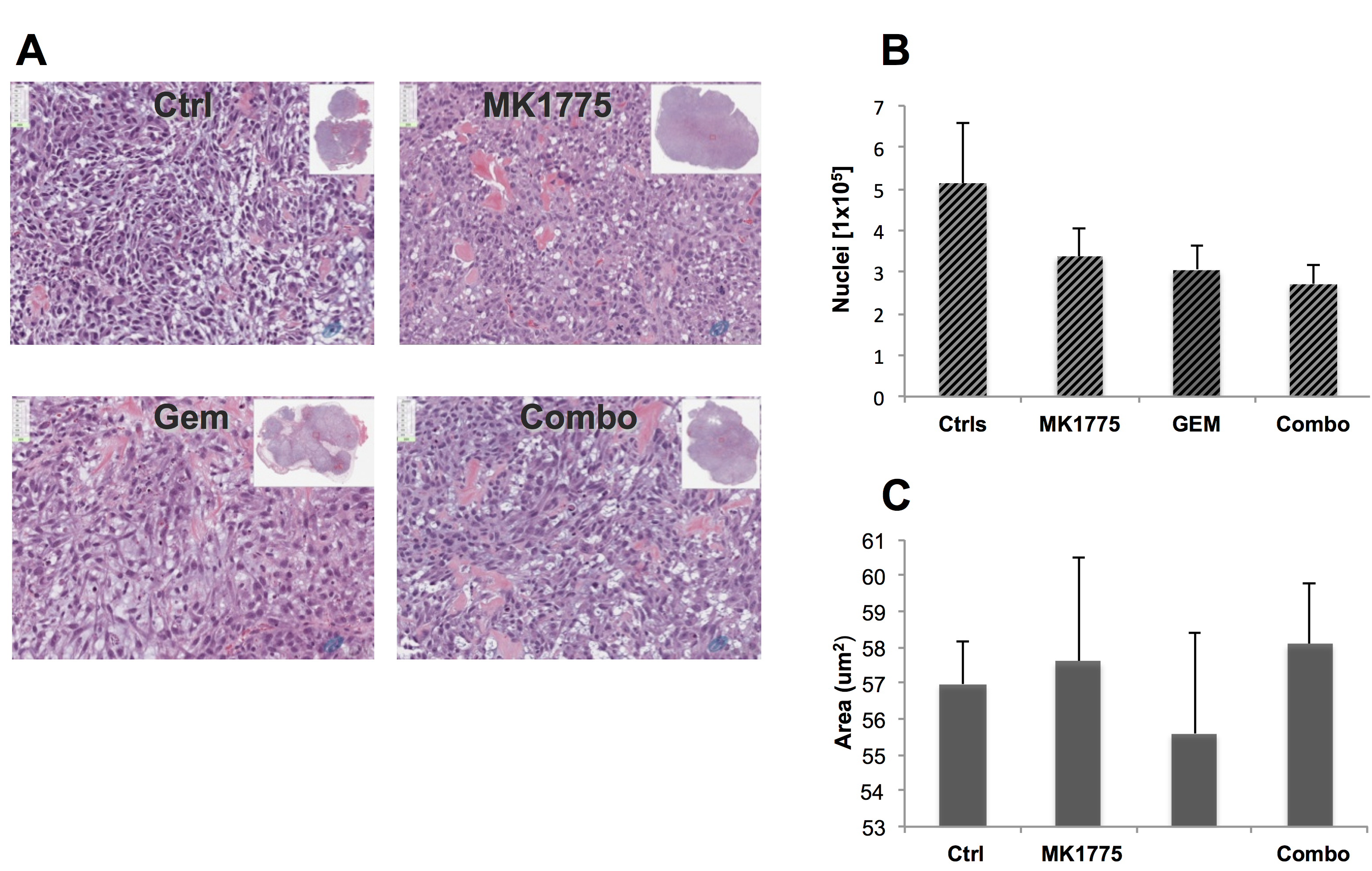

Supplement: Figure S1 — Visual and quantitative analysis of standard H&E sections herein displayed with 10X zoom. (A) H&E sections from each group were analyzed for potential bone formation, necrosis, number of nuclei and average cell area. (B) Cell number analysis across groups indicates a trend of fewer cells in the treated groups compared to controls and. (C) Quantification of average cell area for each tumor and group also indicates a larger range in cell sizes for the treated groups. (TIFF) [file pone.0082875.s001.tiff]
